# Enhanced Bulbar Function in Amyotrophic Lateral Sclerosis: The Nuedexta Treatment Trial

**DOI:** 10.1007/s13311-016-0508-5

**Published:** 2017-01-09

**Authors:** Richard Smith, Erik Pioro, Kathleen Myers, Michael Sirdofsky, Kimberly Goslin, Gregg Meekins, Hong Yu, James Wymer, Merit Cudkowicz, Eric A. Macklin, David Schoenfeld, Gary Pattee

**Affiliations:** 1 Center for Neurologic Study, 7590 Fay Ave., Suite 517, La Jolla, CA 92037 USA; 2 Cleveland Clinic, Department of Neurology, Mail Code S90, 9500 Euclid Ave., Cleveland, OH 44195 USA; 3 Georgetown University Hospital, Department of Neurology, Pasquerilla Healthcare Center, 7th Floor, 3800 Reservoir Road, N.W., Washington, DC 20007 USA; 4 Providence ALS Center, 5050 NE Hoyt St., #315, Portland, OR 97123 USA; 5 Department of Neurology, University of Minnesota, 420 Delaware St. SE, Minneapolis, MN 55455 USA; 6 Neurological Clinical Research Institute and Biostatistics Center, Massachusetts General Hospital, 15 Parkman Street, Boston, MA 02114 USA; 7 Harvard Medical School, 25 Shattuck St, Boston, MA 02115 USA; 8 Department of Neurology, University of Florida College of Medicine, HSC P.O. Box 100236, Gainesville, FL 32610-0236 USA; 9 Neurology Associates, 2631 S. 70th St., Lincoln, NE 68506 USA

**Keywords:** Amyotrophic lateral sclerosis, bulbar function, Nuedexta, dextromethorphan, self-report scale, clinical trial

## Abstract

The goal of this randomized, blinded, crossover clinical trial was to determine whether Nuedexta (dextromethorphan and quinidine) enhanced speech, swallowing, and salivation in patients with ALS. Sixty patients with amyotrophic lateral sclerosis (ALS) received either Nuedexta or placebo for 28 to 30 days, followed by a 10 to 15-day washout period. Subsequently, patients were switched to the opposite treatment arm for the remaining days of the trial. The primary endpoint was a reduction in the self-report Center for Neurologic Study Bulbar Function Scale (CNS-BFS) score. The rater-administered ALS Functional Rating Scale Revised was the principal secondary endpoint. The CNS-BFS score improved with active treatment, decreasing from a mean of 59.3 in the placebo arm of the trial to 53.5 during the drug-treatment arm (*p* < 0.001). Each of the individual domains of bulbar function interrogated by the CNS-BFS responded to treatment with Nuedexta as follows: salivation: 15.8 *versus* 14.3 (*p* = 0.004); speech: 24.6 *versus* 22.2 (*p* = 0.003); swallowing: 18.9 *versus* 17.1 (*p* = 0.009). Similarly, the bulbar component of the ALS Functional Rating Scale Revised improved with active treatment (*p* = 0.003), although the drug did not affect the motor and respiratory components of this scale. This study is unique for several reasons. Firstly, it was driven by patient reports of improved speech and swallowing while taking Nuedexta for control of emotional lability. Secondly, the study was conducted over a short duration (70 days), and thirdly, a self-report scale was selected as the principle outcome measure. Considering the importance of bulbar functions, these results, if confirmed, point to an additional use of Nuedexta as an adjunct to the management of ALS.

**Electronic supplementary material:**

The online version of this article (doi:10.1007/s13311-016-0508-5) contains supplementary material, which is available to authorized users.

## Introduction

To date, palliative care has been the mainstay of treatment for bulbar symptoms that account for much of the disability that accompanies motor neuron disease [1–4]. For example, percutaneous gastrostomy is recommended for patients who are unable to maintain their weight or swallow effectively. On this background, it was unexpected when patients treated with Nuedexta (DMQ), approved in 2011 for the treatment of labile emotionality (pseudobulbar affect) that occurs in association with amyotrophic lateral sclerosis (ALS) [5, 6], reported improvements in speech, swallowing, and the ability to handle oral secretions.

DMQ contains both dextromethorphan (DM) and quinidine. The latter protects DM from O-demethylation by inhibiting the cytochrome P450 isoenzyme CYP2D6 [7]. As is true for many other psychoactive drugs, DM exhibits molecular promiscuity [8]. First recognized as a weak uncompetitive antagonist of *N*-methyl-D-aspartate receptors [9], DM was subsequently discovered to be a sigma-1 receptor agonist [10]. These inhibit voltage gated-ion channels, potentiate ligand-gated channels [11], and exert a modest effect on serotonin uptake [12]. These properties have suggested the drug may be useful for a variety of clinical applications [13, 14].

To us, the clue suggesting that a drug that palliates pseudobulbar affect (PBA) might enhance bulbar function was provided by Parvizi et al. [15], who postulated that the brainstem and cerebellum are involved in the regulation of emotional expression. As sigma-1 receptors are preferentially localized to these structures [16], and recently found to decorate brainstem motor neurons, we felt it reasonable to expect that DMQ, a sigma-1 agonist, might also palliate speech and swallowing, as well as PBA.

For the most part, ALS drug trials have focused on survival as an endpoint. These studies typically utilize a parallel design and have extended for long intervals, often up to 18 months. With one exception [17], these have failed, whereas studies that focused on symptomatic treatment, such as the use of bi-level positive airway pressure for treatment of respiratory failure, have demonstrated a survival benefit associated with an enhanced quality of life [18]. Accordingly, we thought it important to confirm the palliative effect of DMQ on bulbar symptoms as reported by patients, family members, and physicians.

As the clinical assessment of bulbar function has not yet attained the standard of practice that is universally employed for the assessment of limb musculature and respiratory function, we set out to develop a self-report bulbar function scale, the Center for Neurologic Study Bulbar Function Scale (CNS-BFS). This was modeled after the Center for Neurologic Study Emotional Lability Scale (CNS-LS) that has proven to be a robust endpoint in 4 clinical trials [5, 19–21]. The CNS-BFS interrogates 3 domains of bulbar function: speech, swallowing, and salivation (see Table 1). For each domain, patients are asked to rate 7 statements or questions on a scale of 1 to 5. Patients unable to speak are assigned a value of 6 for the speech domain questions. This allows for a global score ranging from 21 to 112. The principal goal of this study was to determine whether DMQ exerts a palliative effect on speech, swallowing, and salivation in patients with ALS, measured in the aggregate by the CNS-BFS, and by the use of quantitative measures, such as speech and swallowing rate.

**Table 1:** Center for Neurologic Study bulbar function scale (CNS-BFS)

**Sample Question:** **Speech**						
	Does not apply 1	Applies rarely 2	Applies occasionally 3	Applies frequently 4	Applies most of the Time 5	Unable to communicate by speaking 6*
1. My speech is difficult to understand.	○	○	○	○	○	○
*Rating 6 only applies to speech						
**Bulbar function domains**						
Salivation		Speech			Swallowing	
1. Excessive saliva is a concern to me. 2. I take medication to control drooling. 3. Saliva causes me to gag or choke. 4. Drooling causes me to be frustrated or embarrassed. 5. In the morning I notice saliva on my pillow. 6. My mouth needs to be dabbed to prevent drooling. 7. My secretions are not manageable.		1. My speech is difficult to understand. 2. To be understood I repeat myself. 3. People who understand me tell other people what I said. 4. To communicate I write things down or use devices such as a computer. 5. I am talking less because it takes so much effort to speak. 6. My speech is slower than usual. 7. It is hard for people to hear me.			1. Swallowing is a problem. 2. Cutting my food makes it easier to chew and swallow. 3. To get food down I have switched to a soft diet. 4. After swallowing I gag or choke. 5. It takes longer to eat. 6. My weight is dropping because I can’t eat normally. 7. Food gets stuck in my throat.	

The patient self-report CNS-BFS interrogates 3 domains of bulbar function: speech, swallowing, and salivation. For each domain, patients are asked to rate 7 statements or questions on a scale of 1 to 5. Patients unable to speak are assigned a value of 6 for the speech domain questions. Scores can therefore range from a low of 21 (no symptoms of bulbar dysfunction) to a high of 112

## Methods

### Study Design and Participants

This phase II, multicenter, double-blind, randomized crossover trial was designed to evaluate the effect of DMQ treatment on bulbar functions (speech, swallowing, and salivation) in patients with ALS.

Sixty patients were recruited from 7 sites chosen by the Northeast ALS Consortium. Eligible participants were at least 18 years old with a diagnosis of probable or definite ALS as defined by the revised World Federation of Neurology El Escorial criteria, disease duration < 2 years from time of diagnosis, bulbar dysfunction manifested by dysarthria and/or dysphagia as determined by the site principal investigator, guided by a CNS-BFS score ranging from 50 to 80.

Further requirements included a slow vital capacity of 50% of normal or greater, intact cognitive function, again determined by the principal investigator, as well as relatively sound general health based on a physical examination and baseline laboratory values obtained at a screening visit. If patients were taking riluzole, they had to have been on the drug for at least 30 days prior to randomization, and, similarly, patients taking medication(s) to control salivation had to be on a stable dose for 30 days prior to be eligible for inclusion in the study.

Exclusion criteria included the following: prior use of DMQ; current use of dextromethorphan, quinidine, quinine, mefloquine, opioids, or a known sensitivity to those drugs; a history of prolonged QT interval, congenital long QT syndrome, complete atrioventricular block, or concomitant use of drugs that both prolong QT interval and are metabolized by CYP2D6; use of monoamine oxidase inhibitors; invasive ventilator dependence; use of a feeding tube; treatment with Botox or radiation for control of sialorrhea within 90 days of screening in the former instance and 180 days in the latter instance.

The trial was approved by the institutional review board at each study site, and informed written consent was obtained from all patients. A medical monitor was available to resolve issues that could affect patient care, their eligibility for enrollment in the trial, or continued participation of the patients in the instance of an adverse event. The study (ClinicalTrials.gov identifier NCT01806857) was conducted in accordance with the ethical principles of the Declaration of Helsinki.

### Randomization and Masking

Patients meeting the eligibility criteria and accepted into the study were randomized in a 1:1 ratio to treatment arms that began with either placebo or DMQ dosing. Study patients, site investigators, and all other study staff including project and data management personnel and the study sponsor were blinded to treatment assignment throughout the study. Placebo and DMQ were provided by the sponsor (Avanir Pharmaceuticals) in identical blister packs and were indistinguishable from one another. Both drug and placebo were securely stored under the recommended conditions at a pharmacy at the University of Rochester. Research pharmacists who prepared and shipped drug supplies to each of the clinical research sites were unblinded as to individual drug assignments in the study.

### Procedures

**Fig. 1:**
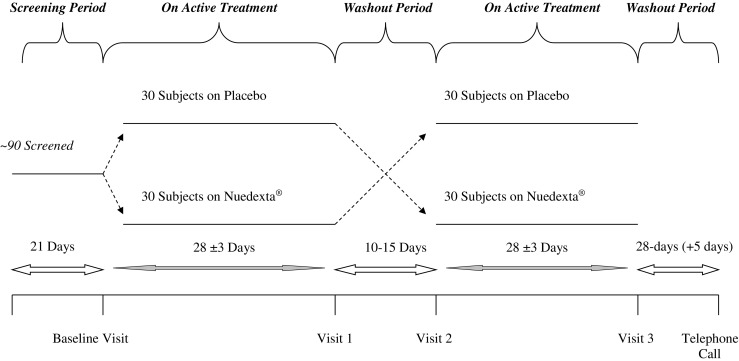
Study design

The 2 treatment arms included placebo and DMQ, a drug known to have a rapid onset of action. Accordingly, each arm of the trial was designed to be 28 to 30 days in duration, separated by a 10 to 15-day washout period (see Fig. 1). At the outset of each treatment period, patients took either a placebo or DMQ capsule in the evening for 7 days. Subsequently, they were instructed to take 2 capsules per day at 12-h intervals for the remainder of the 28-day period. Following the washout period, patients were switched to the opposite treatment arm, with an identical dosing regimen. Nuedexta (DMQ) is a combination product containing 20 mg dextromethorphan hydrobromide and 10 mg quinidine sulfate.

As DMQ is an approved drug with an excellent safety profile [22], baseline laboratory studies were limited to a complete blood count, standard chemistry panel, and an electrocardiogram. The occurrence of adverse events was documented at each study visit, as well as at a final follow-up telephone call. At the beginning and end of each treatment arm patients were administered the test battery, outlined in the next section, to assess their bulbar function.

### Outcome Measures

The primary study objective was to determine whether DMQ exerts a palliative effect on speech, swallowing, and salivation in patients with ALS, as determined by a significant change in the CNS-BFS score. The CNS-BFS is a 21-item self-report scale that assesses 3 domains of bulbar function: speech, swallowing, and salivation (Table 1). The scale was modeled on the CNS-LS that has been a robust endpoint in 4 clinical trials. Initially the CNS-BFS was validated in a large population of ALS patients (*n* = 122) recruited online using a social networking site [23]. Subsequently, the scale was validated in 120 patients consisting of the 60 participants in the main study and an additional 60 individuals selected from the general ALS clinical population recruited from 4 of the 7 participating ALS research centers.

In the instance of the online study, queries for each of the 21 items that comprise the CNS-BFS were compared with the patient’s self-reported visual analog scale scores for speech, swallowing, and salivation. In the subsequent study, the validation was conducted in a clinic setting, the advantage being that the patient’s speech, swallowing, and ability to handle oral secretions were objectively rated as normal or abnormal by a clinician. For example, in the instance of speech, evaluators determined the character of the patient’s speech based on 3 criteria: loudness, intelligibility, and the presence or absence of nasality. This assessment was made during a formal timed reading test. This was an objective assessment in that it did not require any further input from the patients. A similar strategy was employed to objectively assess the patient’s swallowing and ability to manage oral secretions.

At baseline, the CNS-BFS total score was well correlated with the ALS Functional Rating Scale Revised (ALSFRS-R) bulbar subscale (*r* = –0.90, *p* < 0.001). Additionally, the CNS-BFS speech subscale was highly predictive of clinician assessment of impaired speech [area under the curve (AUC) = 0.95, *p* < 0.001]. Similarly, the CNS-BFS swallowing subscale was well correlated with clinician assessments (choking, spillage, abnormal effort; AUC = 0.83, *p* < 0.001), and the CNS-BFS salivation subscale correlated well with clinician assessments (resting or stimulated drooling or dabbing; AUC = 0.88, *p* < 0.001).

Patients were required to complete the self-report CNS-LS scale to assess the occurrence of emotional lability, also known as PBA. This was done because it is known that PBA occurs more commonly in patients with ALS with bulbar symptoms [2, 8], and because DMQ is highly effective in treating PBA. Accordingly, it was of interest to compare the effect of treatment in both groups, that is, patients with and without PBA.

Secondary outcome measures included changes in the self-administered visual analog scales for bulbar function, the Ashworth spasticity scale, the timed reading of a test paragraph, the timed swallowing of both solids and liquids, an observed salivation assessment (both resting and stimulated), and the rater-administered ALSFRS-R. The latter is an ordinal rating scale used to determine patient’s capability and independence in 12 functional activities, all of which are relevant in ALS [24]. The ALSFRS-R has been shown to correlate with changes in strength over time, closely associates with quality of life measures, and predicts survival.

Primary safety variables included monitoring adverse events (AEs), as well as any treatment discontinuations due to them. Secondary safety variables included vital signs, as well as concomitant medication requirements. Compliance was monitored using pill counts in each period.

### Statistical Analysis

In a prior study, DMQ was demonstrated to substantially improve PBA [19].

Accordingly, it was assumed that the effect on speech and swallowing would be of similar magnitude. Based on this, the crossover treatment design that was modeled on the prior study predicted an 88% probability that the study would detect a treatment difference at a 2-sided 0.05 significance level.

The primary efficacy analysis was based on the CNS-BFS score measured at baseline and at the end of each of the 2 study treatment periods for all patients. The data were analyzed using an analysis of covariance with terms for baseline value, treatment group, and period sequence. Patients’ mean CNS-BFS scores were modeled as a random effect. Estimates were calculated by least-squared means with SEs. By including a period term, the model adjusted for changes in symptoms over the course of the study. We tested for a period–treatment interaction to allow for a carry-over effect. Carry-over could occur as a result of a curative effect of therapy or an unblinding of patients on treatment due to efficacy or adverse treatment effects. This analysis assumes that missing data were missing at random, conditional on the model and observed data.

The analysis plan for secondary variables was the same as that for the primary variable. In addition, the percentage of patients whose speech, swallowing, or salivation was palliated with treatment was determined by calculating the number of patients who responded to therapy as a fraction of the total number of patients treated. Lastly, treatment effects on the 3 domains of bulbar function were assessed.

## Results

**Fig. 2:**
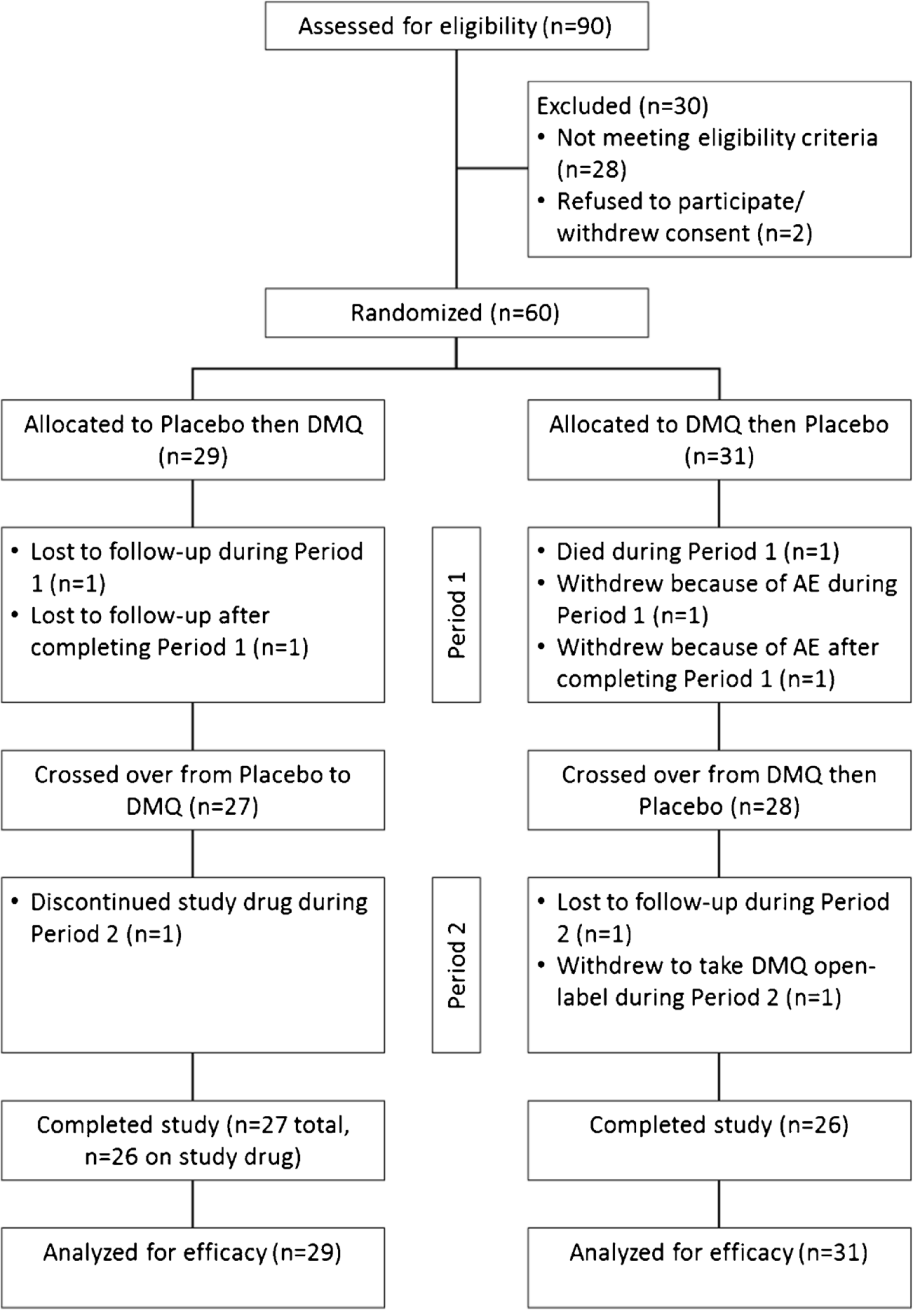
CONSORT diagram. DMQ = Nuedexta; AE = adverse event

From April 2013 to November 2014, 90 patients were screened, 60 of whom were subsequently randomized into the study. Selected baseline characteristics of patients are shown in Table 2. A total of 53 patients completed both arms of the study, one of whom had discontinued study drug during period 2 (see Fig. 2 for the trial profile). One participant died during period 1; 2 withdrew consent owing to AEs and 1 in order to take DMQ open-label; and 3 participants were lost to follow-up. Upon completion of the trial, each site was visited by a clinical research administrator who reviewed the case report forms. Subsequently, all “queries” were resolved and the electronic database for the study was cross-checked to assure that it mirrored the data generated at the trial sites. On 10 July 2015 the database was locked and the results of the study were analyzed following the data analysis format specified in the trial protocol.

**Table 2:** Study randomization and demographics

Category	Patients (*n*)	%
Randomization		
Active then placebo	31	51.7
Placebo then active	29	48.3
Sex		
Female	26	43.3
Male	34	56.7
Race		
Asian	1	1.7
Black/African	2	3.3
White/Caucasian	57	95.0
Ethnicity		
Non-Hispanic or Latino	60	100
Patients taking riluzole	21	35.0
Limb onset	22	36.7
Bulbar onset	38	63.3
Baseline values		
Age (y)	57.8 ± 11.1	
Age range (y)	26–78	
CNS-BFS total score	58.2 ± 13.4	
ALSFRS-R total score	34.6 ± 7.0	
Mean time symptom onset to trial enrollment (mo)	23.3 ± 21.6	
Mean time ALS diagnosis to trial enrollment	9.2 ± 13.3	

Data are mean ± SD unless otherwise indicated. CNS-BFS = Center for Neurologic Study Bulbar Function Scale; ALSFRS-R = Amyotrophic Lateral Sclerosis Function Rating Scale Revised; ALS = Amyotrophic Lateral Sclerosis

**Fig. 3:**
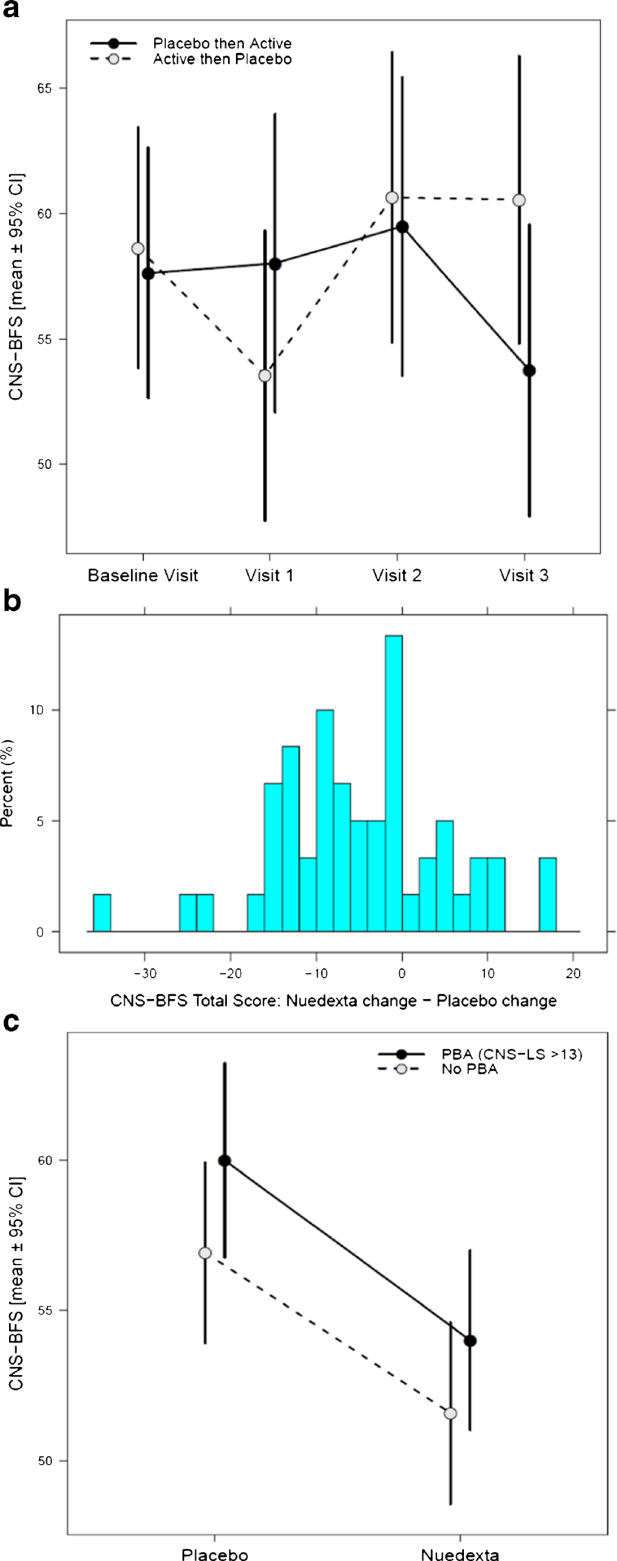
Effect of Nuedexta (DMQ) *versus* placebo on changes in the Center for Neurologic Study Bulbar Function Scale (CNS-BFS; primary outcome measure). (A) Mean CNS-BFS scores for each treatment arm (i.e., patients treated with placebo first and then switched to DMQ *vs* patients placed on DMQ initially and later switched to placebo) were calculated during the course of the clinical trial. Measurements were obtained at baseline, and during 3 subsequent clinical visits. For the group treated with placebo initially, DMQ treatment was initiated following visit 2 and the drug treatment effect measured at visit 3. For the group treated with DMQ initially, drug treatment began immediately following the baseline visit and the effect of treatment was measured at visit 1. The crossover effect is apparent: CNS-BFS scores declined following the period of DMQ treatment. (B) Histogram of unadjusted treatment-dependent change in CNS-BFS total scores among completers. Negative values indicate larger reductions in CNS-BFS total scores after receiving DMQ. Positive values indicate larger reductions after receiving placebo. (C) Interaction plot of CNS-BFS *versus* pseudobulbar affect (PBA) status. Improvement in bulbar function (CNS-BFS) associated with DMQ treatment stratified by presence or absence of PBA at baseline, defined by a score > 13 on the Center for Neurologic Study Emotional Lability Scale (CNS-LS). Mean baseline-adjusted CNS-BFS total scores ± 95% confidence intervals (CI) are displayed on the *y*-axis. DMQ treatment improved bulbar functioning irrespective of whether patients had PBA

Most patients (93%) were > 90% compliant taking study drug, based on pill counts (excluding 2 patients with missing data). The CNS-BFS was, a priori, chosen as the primary endpoint. An intent-to-treat analysis and both center and period effects were determined. There were no center or period effects. For all relevant assessments, comparisons were made between trial periods. For the primary outcome measure, the mean CNS-BFS for the placebo arm of the trial was 59.3 (SE 1.10) *versus* 53.5 (SE 1.07) for the active treatment period (*p* < 0.001). Figure 3(A) illustrates the effect on mean CNS-BFS scores of crossing patients on placebo to the treatment arm and, conversely, switching patients on DMQ to the placebo condition. Figure 3(B) shows that almost twice as many patients had an improvement in their CNS-BFS scores while on DMQ than when they were on placebo. The degree of change is also illustrated in the figure.

Each domain of bulbar function in the CNS-BFS responded positively to DMQ treatment, as follows: 1) salivation 15.8 *versus* 14.3 (*p* = 0.004); 2) speech 24.6 *versus* 22.2 (*p* = 0.003); 3) swallowing 18.9 *versus* 17.1 (*p* = 0.009).

DMQ treatment also resulted in a significant improvement in the bulbar component of the more commonly used, rater-administered ALSFRS-R (*p* = 0.003). Approximately half of the patients improved 1 or more points on this measure. The motor and respiratory components of the ALSFRS-R, however, were not significantly affected by active treatment. Treatment positively affected the speech component of the VAS (*p* = 0.005), but had no significant effect on the swallowing or sialorrhea components of this measure (see Table 3 for a complete summary of primary and secondary efficacy endpoints).

**Table 3:** Primary and secondary efficacy endpoints

Measure	Active Mean	Placebo Mean	Mean Difference Active Minus Placebo	Mean Difference SE	Rx Effect *p*-value
CNS-BFS Total	53.45	59.31	–5.85	1.49	<0.001
CNS-BFS Sialorrhea	14.28	15.81	–1.52	0.51	0.004
CNS-BFS Speech	22.22	24.57	–2.35	0.74	0.003
CNS-BFS Swallowing	17.14	18.92	–1.77	0.65	0.009
VAS Speech	4.97	4.11	0.86	0.30	0.005
VAS Swallowing	7.23	6.93	0.30	0.42	0.47
VAS Sialorrhea	6.78	6.78	–0.01	0.45	0.99
ALSFRS-R Total	34.15	33.70	0.45	0.38	0.25
ALSFRS Bulbar	7.39	6.79	0.60	0.19	0.003
ALSFRS Motor	16.63	16.8	–0.16	0.22	0.46
ALSFRS Respiratory	10.12	10.10	0.02	0.17	0.90
#words read/min	107.12	103.37	3.75	2.54	0.15
Avg swallow water time (sec)	12.16	13.11	–0.95	0.96	0.33
Avg swallow solids time (sec)	18.53	19.45	–0.92	1.66	0.58
Ashworth spasticity scale Score Right Arm	1.65	1.53	0.12	0.09	0.19
Ashworth spasticity scale Score Left Arm	1.62	1.67	–0.05	0.09	0.58
Ashworth spasticity scale Score Right Leg	1.94	1.82	0.11	0.07	0.10
Ashworth spasticity scale Score Left Leg	1.91	1.91	0.00	0.08	0.97
CNS Lability Scale Total	10.79	13.72	–2.92	0.68	<0.001

Mean values in patients’ Center for Neurologic Study Bulbar Function Scale (CNS-BFS) scores (the primary efficacy endpoint) and various secondary efficacy endpoints are depicted following completion of the placebo and the Nuedexta (DMQ) arms of the trial. The mean differences in patients’ placebo treatment scores subtracted from their DMQ treatment scores are shown, along with SEs of these mean differences, and *p*-values of the treatment effect. Note that statistically significant improvements as a result of DMQ treatment were observed for all 3 components of the primary outcome measure, the CNS-BFS, as well as the visual analog scale (VAS) speech scale and the bulbar component of the Amyotrophic Lateral Sclerosis Functional Rating Scale Revised (ALSFRS-R). As expected, DMQ treatment also resulted in improvements in scores measuring inappropriate emotionality [Center for Neurologic Study Emotional Lability Scale (CNS-LS)]

All of the quantitative measures for speech and swallowing improved during the DMQ arm of the trial, although none of these improvements attained a level of statistical significance. The number of words read per minute increased from 103 in the placebo period to 107 in the treatment period (*p* = 0.15). The time it took to swallow 30 ml of water decreased from 13.1 s in the placebo group to 12.2 s in the treatment group. Similarly, the time to swallow a teaspoon of cereal decreased with DMQ treatment (19.5 s *vs* 18.5 s).

As expected, treatment had a robust effect on inappropriate emotionality as determined by the CNS-LS (13.7 placebo *vs* 10.7 treated; *p* < 0.001). At the outset, it was not certain that DMQ treatment would palliate impaired speech and swallowing in patients who did not exhibit pseudobulbar affect. However, this was the case, as illustrated in Figure 3(C), which shows that patients with and without PBA experienced an equivalent improvement in their total CNS-BFS scores following DMQ treatment. There was no significant correlation between the baseline CNS-LS score and the response to treatment (*r* = –0.054, *p* = 0.70).

Patients were monitored for adverse events during both arms of the study, most of which were mild or moderate in nature (see Table 4). The most frequent side effects reported during use of DMQ were constipation, diarrhea, nausea, and dizziness, similar to results reported in previous trials evaluating DMQ for the treatment of emotional lability [5]. AEs reported in > 5% of patients during treatment and placebo intervals of the trial are listed in Table 5.

**Table 4:** Adverse event summary by severity and relationship to study drug

	DMQ (*n* = 58)			Placebo (*n* = 57)		
Adverse event characteristic	# of Events	# of Subjects	% of Subjects	# of Events	# of Subjects	% of Subjects
Severity						
No AEs reported	0	32	55%	0	32	56%
Mild	52	15	26%	32	10	18%
Moderate	17	9	16%	16	12	21%
Severe	2	2	3%	3	3	5%
Relationship to Study Drug						
No AEs reported	0	32	55%	0	32	56%
Not related	18	5	9%	30	13	23%
Unlikely related	28	12	21%	14	8	14%
Possibly related	15	6	10%	7	4	7%
Probably related	10	3	5%	0	0	0%

The number and severity of all adverse events (AEs) occurring during placebo and Nuedexta (DMQ) intervals of the trial are listed, along with their likelihood of being related to study treatment. Counts and percentages of patients summarize the most severe or most closely related event reported for each patient during a given treatment interval

**Table 5:** Adverse event summary by MedDRA system organ class, preferred term, and treatment

	DMQ (*n* = 58)			Placebo (*n* = 57)		
MedDRA System organ class and Preferred term	# of Events	# of Subjects	% of Subjects	# of Events	# of Subjects	% of Subjects
Gastrointestinal Disorders						
Constipation	5	5	9%	2	2	4%
Diarrhea	5	5	9%	1	1	2%
Nausea	5	4	7%	0	0	0%
Nervous System Disorders						
Dizziness	10	7	12%	1	1	2%

Adverse events occurring in greater than 5% of subjects during treatment and placebo intervals of the trial are listed. (MedDRA—Medical Dictionary for Regulatory Activities)

Five patients in the trial experienced severe AEs, 2 of them during the DMQ arm of the trial and 3 patients during the placebo arm of the trial. One patient died of respiratory failure secondary to ALS while on the DMQ arm of the trial. Another patient had to be discontinued from DMQ therapy because of recurring nausea and diarrhea. The number of patients experiencing AEs during both arms of the trial and the degree to which the AEs are believed related to study treatment are depicted in Table 4. There were no clinically relevant changes in vital signs from baseline through the final visit in the study. This safety profile is similar to that recently reported in a multicenter study to assess the safety of DMQ [25].

## Discussion

Considering past ALS treatment trials, this study is unique for several reasons. Firstly, it was driven by reports from patients and family members who, having been treated for one condition (PBA), reported unexpected benefit upon seemingly unrelated symptoms. Secondly, the study was conducted over a relatively short time period (70 days) and, thirdly, a self-report scale was selected as the principle outcome measure. So far, most ALS studies have focused on slowing disease progression. This is the first controlled study to report an improvement of bulbar function, specifically the enhancement of speech and swallowing, and improved ability to handle oral secretions. It had been anticipated that patients’ PBA status could be a treatment variable. This proved not to be the case. Patients with and without inappropriate emotionality both responded to treatment (see Fig. 3C).

While the results of this study are statistically robust, one could ask whether the result is clinically meaningful. Few data have been published as to what would constitute a clinically relevant change in an ALS trial by any measure. Recently, a group of ALS specialists concluded that a 2-point change in the ALSFRS-R was “moderately or very clinically meaningful” in both the gross and bulbar domains of this scale. Germane to this study, the authors stated that “smaller changes in bulbar and respiratory functional domains were considered more clinically relevant than in other domains” [26]. Just under half (49%) of the patients in this study improved by 1 or more points in the ALSFRS-R bulbar domain with DMQ treatment relative to their response on placebo *versus* 26% who experienced improvements on placebo. In short, even by this traditional measure, the results of the study suggest a favorable clinical outcome. In this regard, there has been a longstanding concern that ALS trials, uniformly unsuccessful, might benefit from better assessment tools. Efforts to remedy this, in the instance of bulbar dysfunction, are ongoing [27–30]. Our data strongly suggest that the use of patient-generated information may meet this need. The primary outcome measure in this study, the CNS-BFS, proved to be superior in all instances to test instruments historically utilized in clinical trials: visual analog scales, timed measures of speech and swallowing, and the ALSFRS-R (manuscript in preparation). As observed in this study, not all self-report measures are equally informative. Whereas all components of the CNS-BFS were favorably affected with treatment, only the speech visual analog scale was improved. The reason for this is conjectural. Had this study relied solely on quantitative outcome measures, such as speech rate or timed swallowing, it would have failed to detect a significant treatment effect. While a treatment response might have been evident in a larger study, it is noteworthy that measures such as speech rate do not fully reflect all the nuances of speech. In short, a seemingly objective measure of a function such as speech may be better assessed by a patient than by an arbitrary metric. In the future this limitation may be remedied through the use of computer-assisted assessment of speech. Recordings obtained from patients in this study are currently undergoing analysis.

This study leaves some questions unanswered, as it would have taken a longer study with more patients to do so. The study was not designed to predict the duration of the treatment effect or the impact of treatment on disease progression. A phase III trial to address these considerations is in the planning stage. Nevertheless, there is anecdotal evidence to suggest a longstanding benefit of treatment in some patients. But considering the fact that bulbar onset ALS and bulbar-associated symptoms are regarded as ominous, portending a grave prognosis, it is reasonable to assume that any treatment that ameliorates this aspect of ALS could be a useful addition to the treatment armamentarium [31, 32].

On reflection, it is tempting to consider whether this trial offers any clues as to the mode of DMQ’s treatment effect and any guidance regarding the future design of ALS clinical trials. As previously noted, DMQ exerts pleiotropic pharmacologic effects. Which of its effects, alone or in combination, account for the favorable treatment outcome in this study will require further investigation. We doubt that its effect on glutamate is primarily responsible for at least 2 reasons. Firstly, riluzole has never been reported to enhance bulbar function but, interestingly patients with bulbar-onset disease were reported to be more responsive to treatment than other patients [33]. Secondly, ceftriaxone, a drug that upregulates the glutamate transporter, failed to exert any benefits in a large, controlled study [34]. As sigma-1 receptors preferentially decorate brainstem neurons, the most parsimonious explanation for the effect of DMQ is its ability to facilitate the function of these motor neurons.

It should be noted that 18 of the 52 patients reported the same or worse CNS-BFS scores after DMQ treatment relative to placebo (see Fig. 3B). Nonresponders could represent a subgroup that is refractory to this mode of treatment for one reason or another. In the era of personalized medicine, identification of such a subgroup would be of paramount importance. A parallel argument could be made that patients’ who responded to treatment did so based on a nonspecific drug effect. By analogy with the placebo effect, it might be assumed that a drug such as DMQ that affected patients’ mood or enhanced arousal, to cite 2 factors, could favorably influence patients’ perception of their ability to speak and/or swallow. While we cannot exclude this possibility, we consider it unlikely, primarily because treatment had no effect on patients’ ability to perform a wide range of other activities as interrogated by the ALSFRS-R. Only the bulbar component of the ALSFRS-R significantly changed during the treatment limb of the trial. Patients noted no change in other components of the ALSFRS-R, including their ability to write, dress, climb stairs, and so on.

This trial result could conceivably redirect thinking about future ALS drug trials which, to date, have primarily emphasized survival as the primary endpoint. At the minimum, this trial demonstrates that all motor neurons are not created equal. It has long been known that cranial nerves innervating the eye muscles for the most part are resistant to ALS [35]. As we observed no effect on functions subserved by spinal motor neurons, it is fair to state that these neurons are qualitatively different than the cranial motor neurons that facilitate bulbar functions such as speech and swallowing. Given the success of this trial, one could argue for placing more emphasis in the future on treatments that enhance the functional abilities of patients with ALS and kindred disorders. A step in this direction might be the recent effort to utilize a skeletal muscle activator to enhance motor function [36]. Equally important might be a drug trial to enhance cognitive function in the instance of ALS associated with frontotemporal dementia. Until we have a better understanding of ALS, this treatment strategy may be the fork in the road not yet taken.

## Electronic supplementary material

Below is the link to the electronic supplementary material.

ESM 1 (PDF 1225 kb)
